# Enorme kyste parotidien du premier arc branchial: à propos d´un cas

**DOI:** 10.11604/pamj.2020.37.334.17191

**Published:** 2020-12-10

**Authors:** Ahmed Rouihi, Bouchaib Hemmaoui, Noureddine Errami, Fouad Benariba

**Affiliations:** 1 Service d´Oto-Rhino-Laryngologie et de Chirurgie Cervico-Faciale, Faculté de Médecine et Pharmacie de Rabat, Université Mohamed V de Rabat, Hôpital Militaire d´Instruction Mohamed V de Rabat, Rabat, Maroc

**Keywords:** Appareil branchial, kyste intra parotidien, chirurgie, Branchial apparatus, parotid cyst, surgery

## Abstract

Les kystes branchiaux intra parotidiens sont des malformations congénitales rares et mal connues de la première fente branchiale. Ils se caractérisent par 3 types de manifestations qui peuvent être associées ou isolées, tuméfaction inflammatoire de la région sous-auriculaire et parotidienne inférieure avec ou sans fistule cutanée cervicale se projetant dans l´aire cervico-mandibulaire et une fistule du plancher du conduit auditif externe avec ou sans otorrhée, avec ou sans bride prémyringienne à l'otoscopie. Ils passent souvent inaperçus, en dehors des épisodes de surinfection. Le diagnostic repose sur l´interrogatoire, notion d´épisodes récidivants d´abcès ou de surinfection. Il n´y a pas d´examen complémentaire d´imagerie à réaliser systématiquement. Dans les formes atypiques, l´échographie ainsi que l´imagerie par résonnance magnétique (IRM) peuvent s´avérer nécessaires, notamment dans les formes parotidiennes pour préciser la nature kystique de la tuméfaction. Les abcès et les épisodes de surinfection récidivants sont en effet les complications les plus fréquemment rencontrés. Sur le plan thérapeutique, l´exérèse chirurgicale est indiquée de façon systématique. Nous rapportons dans cette observation le cas d´un patient ayant un énorme kyste branchial de localisation parotidienne.

## Abstract (en)

Parotid branchial cysts are rare and poorly known congenital malformations of the first branchial cleft. They are characterized by three types of manifestations which may be associated to it or isolated, including inflammatory swelling of the subauricular and inferior portion of the parotid with or without cervico-cutaneous fistula projecting into the cervico-mandibular area and a fistula in the floor of the external auditory canal with or without otorrhea, with or without pretimpanic bridle on otoscopy. They often go unnoticed, except for superinfections. Diagnosis is based on interview, the occurrence of recurrent abscesses or superinfections. No complementary imaging examination should be systematically performed. In atypical forms, ultrasound as well as magnetic resonance imaging (MRI) could be necessary, particularly in patients with parotid swellings, in order to confirm cystic structure. Abscesses and recurrent superinfections are the most common complications. Treatment is based on surgical resection. We report the case of a patient with voluminous parotid cyst of the pharyngeal arch.

## Introduction

Les kystes branchiaux de la parotide sont des malformations dysembryologiques rare à expression auriculo branchiale. Ils sont dus à un défaut de migration et de coalescence de bourgeons embryonnaires de la première fente branchiale [1].

## Patient et observation

**Figure 1:**
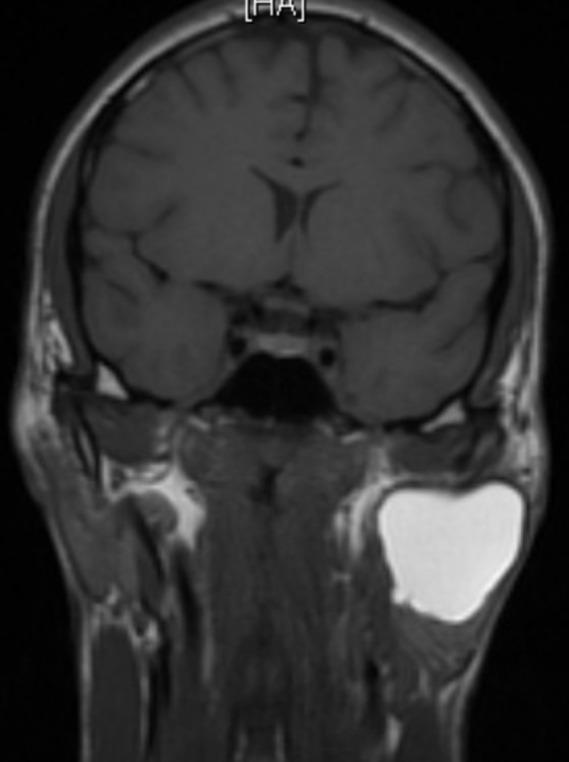
IRM parotidienne coupe coronale montrant un kyste branchial de localisation intra-parotidienne

**Figure 2:**
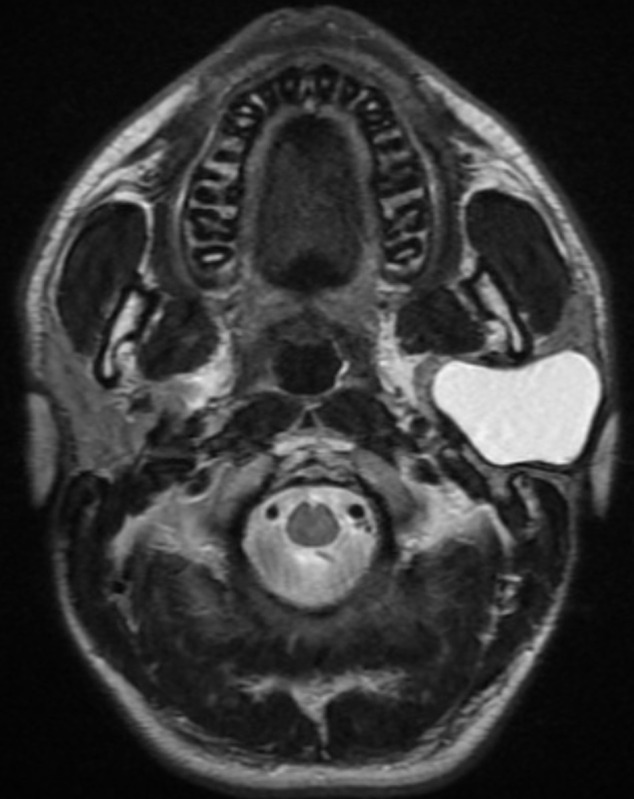
IRM parotidienne coupe axiale montrant un kyste branchial de localisation intra-parotidienne

Mr A.I. âgé de 21 ans, sans antécédents pathologiques notables, qui présente depuis l´enfance une tuméfaction de la région parotidienne gauche, qui a augmenté progressivement de volume. L´examen clinique a retrouvé une tuméfaction de la région parotidienne gauche qui faisait 12 cm de grand axe indolore, de consistance molle, mobile par rapport aux deux plans avec un aspect de la peau sain en regard. L´examen de la cavité buccale et de l´oropharynx n´a pas objectivé de bombement parapharyngé. L´examen otoscopique a mis en évidence une fistule au niveau du plancher du conduit auditif externe homolatéral. Par contre, le patient n´avait pas de trismus ni paralysie faciale. Les aires ganglionnaires cervicales étaient libres. Le reste de l´examen oto-rhino-laryngologique et somatique était normal. Le patient a bénéficié d´une échographie cervicale qui a objectivé la présence d´une formation kystique aux dépens de la glande parotide gauche faisant 10 cm de grand axe. Une IRM parotidienne a été demandée, a mis en évidence une formation bien limitée de la parotide gauche avec un signal bas en T1 et élevé en T2 avec des parois fines et invisibles, qui deviennent épaissies et rehaussées par l´injection de produit de contraste (PDC), (Figure 1 et Figure 2). Le patient a bénéficié d´une parotidectomie exo-faciale. Les suites postopératoires immédiates étaient simples. L´examen histologique définitif a confirmé l´origine embryonnaire branchiale du kyste. L´évolution était favorable avec une absence de récidive après un an.

## Discussion

Les kystes intra parotidiens d´origine branchiale sont des kystes malformatifs dysembryologiques dus à une anomalie d´accolement de la partie ectodermique de la première fente responsable de la genèse des kystes et fistules auriculo-branchiaux. Ils représentent entre 1-8% des anomalies congénitales du premier arc branchial et 1% des anomalies cervicales [1]. Hunczowski décrit la première fente branchiale en 1789. Toutefois, le premier traitement chirurgical d'un kyste branchial n´a été rapporté qu´en 1859 par Langenbeck. Néanmoins, le premier kyste branchial de la glande parotide a été décrit par Hildebrandt en 1895. Ces malformations touchent le plus souvent l´enfant, avant l´âge de cinq ans et l´adulte dans la troisième décade, avec un âge moyen de 44 ans et un ratio homme-femme de 2: 1 [2]. Il existe deux types anatomiques selon les rapports avec la glande parotide et surtout avec le nerf facial selon la classification d´Arnot en 1971 [3]:

**Le type I**: considéré comme une duplication du conduit auditif externe (CAE) membraneux, le kyste siège dans la région rétro auriculaire, le trajet fistuleux se dirige en dedans et en avant parallèlement au CAE et passe en dehors du nerf facial pour s´ouvrir dans la portion osseuse du CAE. L´examen histologique ne montre pas d´annexe cutanée ni résidus cartilagineux, ce qui indique une origine ectodermique.

**Le type II**: plus fréquent que le type I, les lésions associent un kyste de la partie inférieure de la région parotidienne, un trajet fistuleux ascendant passant en pleine glande parotide indifféremment en dedans ou en dehors du nerf facial. L´orifice supérieure est situé à la jonction ostéo cartilagineuse du CAE. L´histologie montre une différenciation à type d´annexes cutanées ainsi que des dérivés cartilagineux.

L´examen clinique retrouve généralement une tuméfaction, indolore, molle et fluctuante de taille variable, généralement de 0,5 cm à 5 cm de diamètre, siégeant le plus souvent dans le lobe superficiel de la glande parotide et notamment dans sa partie inférieure. L´orifice externe de la fistule est situé dans le triangle de Poncet et l´orifice interne est situé sur le plancher du CAE, le trajet de la fistule est parallèle au CAE, en dehors des épisodes de surinfection qui sont souvent révélatrices, ces lésions passent souvent inaperçus. Les kystes se développent dans la parotide ou dans les ganglions lymphatiques de la glande parotide et sont radiologiquement spécifiques en l´absence de fistule [4]. La bilatéralité est exceptionnelle, on en relève deux cas dans la littérature [5]. Les examens d´imagerie sont de grande valeur diagnostique, l´échographie couplée au Doppler, est un examen de faible coût, demandée en première intention, elle permet le diagnostic positif, le siège et la nature kystique; elle permet de guider une ponction à l´aiguille fine, elle met généralement en évidence une formation arrondie, à contenu anéchogène avec renforcement postérieur, bien limitée, à paroi inexistante ou très fine sans végétation tissulaire dans les formes non abcédées. Le Doppler couleur permet d´identifier les composantes vasculaires d´une masse et de préciser ses rapports avec les vaisseaux du cou. Mais elle ne permet pas l´exploration des structures profondes. La tomodensitométrie (TDM) localise et identifie le kyste qui apparaît arrondi, à contenu homogène, sans prise de contraste, sauf en périphérie. L´IRM reste l´examen de choix, elle montre un kyste ovale ou rond de la parotide, la loge parotidienne ou l´espace para pharyngé, et aussi de préciser la nature kystique de la tuméfaction, et d´apprécier son extension et ses rapports, ils ont un signal bas en T1 et élevé en T2. Leurs parois sont fines, mais peuvent être épaissies et rehaussées par l´injection de PDC lorsque le kyste est surinfecté. La visualisation d´un trajet fistuleux se prolongeant vers le CAE, grâce à des coupes coronales TDM ou en IRM, est très évocatrice du diagnostic [6].

Le diagnostic différentiel est représenté par les autres lésions qui comportent une composante kystique, comme les carcinomes adénoïdes kystiques (hypo signal T1, signal variable T2), les adénopathies nécrotiques malignes, le lymphangiome, le kyste lympho épithélial (HIV), le kyste pré auriculaire (défaut de coalescence des ébauches de l´oreille externe); ainsi toute formation kystique de densité homogène, parotidienne ou juxta-parotidienne doit évoquer un kyste de la 1^èr^^e^ fente branchiale. Sur le plan thérapeutique, l´exérèse chirurgicale est indiquée de façon systématique du fait des complications infectieuses. Elle doit être réalisée le plus tôt possible pour limiter les risques de remaniements inflammatoires liés aux épisodes de surinfection, il conviendra alors de n´opérer qu´à après refroidissement par une antibiothérapie adaptée, l´intimité des rapports des kystes de la première fente avec le nerf facial impose une voie d´abord de parotidectomie avec une résection de l´orifice fistuleux, la fistule est ensuite disséquée de proche en proche en découvrant ses rapports avec le nerf facial, s´ il existe, l´orifice interne sera reséquée avec ablation d´un fragment cartilagineux du plancher du conduit auditif externe [7]. En absence de traitement, les kystes évoluent vers la surinfection, la fistulisation à la peau, le syndrome de compression cervicale, la dégénérescence néoplasique est extrêmement rare [4,8], néanmoins, les complications iatrogènes sont dominées par la lésion du nerf facial et de la carotide et la récidive [8].

## Conclusion

Les kystes du premier arc branchial de localisation parotidienne sont des malformations dysembryologiques rares; dus à un défaut de fermeture de la première fente branchiale, leurs diagnostic est parfois difficile. Le traitement est toujours chirurgical qui doit être réalisé à distance des épisodes infectieux. Les complications post-opératoires sont représentées principalement par la récidive et la paralysie faciale.
